# Infectious Disease Agents Associated with Pulmonary Alterations in Aborted Bovine Fetuses

**DOI:** 10.3390/ani12131596

**Published:** 2022-06-21

**Authors:** Thalita Evani Silva de Oliveira, Gabriela Sanches Scuisato, Juliana Torres Tomazi Fritzen, Denise Correia Silva, Rodrigo Pelisson Massi, Isadora Fernanda Pelaquim, Luara Evangelista Silva, Eduardo Furtado Flores, Renato Lima Santos, Lucienne Garcia Pretto-Giordano, Júlio Augusto Naylor Lisbôa, Amauri Alcindo Alfieri, Selwyn Arlington Headley

**Affiliations:** 1 Laboratory of Animal Pathology, Department of Veterinary Preventive Medicine, Universidade Estadual de Londrina, Londrina 86057-970, Brazil; thalitamvet@uel.br (T.E.S.d.O.); gabriela.sanches@uel.br (G.S.S.); isadorapelaquim@gmail.com (I.F.P.); luara.evangelista@uel.br (L.E.S.); 2 Laboratory of Animal Virology, Department of Preventive Veterinary Medicine, Universidade Estadual de Londrina, Londrina 86057-970, Brazil; jufritzen@uel.br (J.T.T.F.); denise.correia@uel.br (D.C.S.); rodrigo.veterinaria@hotmail.com (R.P.M.); alfieri@uel.br (A.A.A.); 3 Department of Preventive Veterinary Medicine, Universidade Federal de Santa Maria, Santa Maria 97105-900, Brazil; eduardofurtadoflores@gmail.com; 4 Department of Veterinary Clinics and Surgery, Escola de Veterinária, Universidade Federal de Minas Gerais, Belo Horizonte 31270-901, Brazil; rsantosufmg@gmail.com; 5 Laboratory of Veterinary Microbiology and Infectious Diseases, Department of Preventive Veterinary Medicine, Universidade Estadual de Londrina, Londrina 86057-970, Brazil; lgiordano@uel.br; 6 Large Animal Internal Medicine, Department of Veterinary Clinics, Universidade Estadual de Londrina, Londrina 86057-970, Brazil; janlisboa@uel.br; 7 National Institute of Science and Technology for Dairy Production Chain (INCT–LEITE), Department of Preventive Veterinary Medicine, Universidade Estadual de Londrina, Londrina 86057-970, Brazil

**Keywords:** abortion, BoGHV6, BVDV, BRSV, fetopathy, interstitial pneumonia, transplacental infection

## Abstract

**Simple Summary:**

A retrospective study was performed to identify pulmonary alterations and/or pneumonia in aborted bovine fetuses (n = 37) and to associate the presence of infectious disease antigens and nucleic agents with patterns and/or alterations of pulmonary disease. Immunohistochemical (IHC) assays were performed to identify antigens of selected agents associated with bovine respiratory disease: bovine alphaherpesvirus 1 (BoAHV1), bovine viral diarrhea virus (BVDV), bovine parainfluenza virus 3 (BPIV-3), bovine respiratory syncytial virus (BRSV), and *Mycoplasma bovis*. Molecular assays were performed to identify nucleic acids of a panel of reproductive disease agents and bovine gammaherpesvirus 6 (BoGHV6) in the lungs of 12 fetuses. Only interstitial pneumonia (12/37) and suppurative bronchopneumonia (1/37) were observed; pneumonia was not observed in 65% of the tissues evaluated. The most frequent intralesional agents were BRSV (16.2%; 6/37), BVDV (13.5%; 5/37), and BoAHV1 (8.1%; 3/37). Interstitial pneumonia was associated with BRSV (n = 3), BoAHV1 (n = 3), and BVDV (n = 2); suppurative bronchopneumonia contained a Gram-positive bacterium and intralesional antigens of BVDV and BRSV. Nucleic acid detection identified at least one disease agent in 75% of the fetuses examined. Reproductive pathogens detected included *Leptospira* spp., (n = 3), BVDV, *Neospora caninum,* and *Brucella abortus* (n = 2). BoGHV6 DNA was identified in the lungs of two fetuses with interstitial pneumonia. Single (n = 7), dual (n = 3), triple (n = 4), and quadruple (n = 1) mixed infections were identified while infectious agents were not identified in 59.5% (22/37) of the examined lungs. Single fetal infections were associated with BRSV, BVDV (n = 2), *Leptospira* spp., BoAHV1, and BoGHV6 (n = 1). These results indicate that the fetuses suffered intrauterine infection through transplacental transmission. The identification of BRSV and BoGHV6 in multiple fetuses with associated pulmonary alterations warrants further investigation relative to the role of these agents in fetopathy and possible direct and/or indirect effects on fetal survival.

**Abstract:**

This study investigated the occurrence of selected pathogens of bovine respiratory disease in fetal pulmonary tissue of cattle and associated these with patterns of disease. Fetal pulmonary (n = 37) tissues were evaluated by histopathology; immunohistochemical assays identified intralesional antigens of bovine alphaherpesvirus 1 (BoAHV1), bovine viral diarrhea virus (BVDV), bovine parainfluenza virus 3 (BPIV-3), bovine respiratory syncytial virus (BRSV), and *Mycoplasma bovis*. Molecular assays were performed to amplify reproductive disease pathogens and bovine gammaherpesvirus 6 (BoGHV6) from 12 lungs. The 2 patterns of pulmonary diseases were interstitial pneumonia (12/37) and suppurative bronchopneumonia (1/37). The frequency of the intralesional antigens identified was BRSV (16.2%; 6/37), BVDV (13.5%; 5/37), BoAHV1 (8.1%; 3/37), *M. bovis* (5.4%; 2/37), and BPIV-3 (2.7%; 1/37). Interstitial pneumonia was associated with BRSV (n = 3), BoAHV1 (n = 3), and BVDV (n = 2); suppurative bronchopneumonia contained a Gram-positive bacterium and BVDV and BRSV. Reproductive pathogens detected included *Leptospira* spp., (n = 3), BVDV, *Neospora caninum,* and *Brucella abortus* (n = 2). BoGHV6 DNA was identified in the lungs of two fetuses with interstitial pneumonia. These findings suggest that these fetuses were infected transplacentally by several pathogens. The role of some of these pathogens herein identified must be further elucidated in the possible participation of fetal disease.

## 1. Introduction

The bovine respiratory disease (BRD) complex is a multifactorial and multi-etiological disease associated with several bacterial and viral agents, together with risk factors or stressors, that favor the development of pneumonic conditions, resulting in varying rates of morbidity and mortality in cattle of all age groups [1,2,3]. Frequent stressors of BRD include weaning, comingling, transportation, abrupt dietary alterations [1,3], and several management factors at feedlots [1]. In Brazil, information relative to the occurrence of infectious agents associated with BRD is scarce and insipient [4] when compared with the data existing in North America [1,2,5,6] and Australia [5]. Consequently, it is difficult to correlate productive losses due to the BRD in feedlot cattle since the available data may not reflect the real situation of cattle health, as well as morbidity and mortality indices in Brazil [6].

The viral agents frequently associated with BRD include bovine alphaherpesvirus 1 (BoAHV1), bovine viral diarrhea virus (BVDV), bovine parainfluenza virus 3 (BPIV-3), bovine respiratory syncytial virus (BRSV), and bovine coronavirus (BCoV) [7,8,9]. Bacterial agents associated with BRD include *Histophilus somni, Mannheimia haemolytica, Mycoplasma bovis,* and *Pasteurella multocida* [4,8,10,11]. Our group has identified all of these agents in feedlot and dairy cattle with BRD from several geographical regions of Brazil [10,12,13,14,15] and has contributed to the understanding of disease patterns associated with the development of BRD [10,15].

Although numerous reports have investigated the infectious agents associated with BRD in feedlot cattle worldwide [4,7,8,15], there are comparatively fewer studies with histologic details involving fetal lungs of cattle [16,17,18] as compared with the innumerous studies describing the lesions observed in several fetal organs. Infectious agents previously associated with fetal lungs and/or pneumonia in cattle include *Brucella abortus* [19,20], *M. bovis* [17], BPIV-3 [18], BoAHV1 [16,21], and BVDV [10]. Most of these studies have identified the associated agents by in situ diagnostic methods, such as immunohistochemistry (IHC) [10,16,17], in situ hybridization (ISH) [17], as well as molecular identification [16,18] and culture and isolation [19,21] in conjunction with histopathologic evidence of pulmonary disease. The IHC and ISH diagnostic strategies demonstrate the intralesional presence of agent-specific antigens associated with histopathological evidence of lesions [22,23], with the obtained results being a strong indication of an associated disease process within the affected tissues [23], thereby providing evidence of the related disease agent with the pattern of pulmonary disease [15]. Furthermore, diagnostic IHC is recommended to identify a wide range of infectious reproductive agents in cattle [24].

This study investigated the presence of selected infectious agents of BRD in aborted bovine fetal lungs to determine whether these pathogens were associated with pneumonia and/or other pulmonary alterations.

## 2. Materials and Methods

### 2.1. Sample Collection, Study Location, and Inclusion Criteria

Retrospective studies were performed on aborted bovine fetuses submitted for histopathologic diagnosis at the Laboratory of Animal of Pathology, Universidade Estadual de Londrina (UEL), Paraná, southern Brazil, and at the Veterinary Diagnostic Laboratory, Universidade Federal de Minas Gerais (UFMG), midwestern Brazil, from 2009 to 2019.

All files within the registry were reviewed to identify fetal bovine tissues submitted for diagnosis. Subsequently, only cases that contained the pathologic data and the correlated paraffin blocks and/or glass slides of fetuses with pulmonary tissue were included in this study. Additionally, when necessary, histological slides were redone by using the Hematoxylin and eosin staining technique. Furthermore, the Giemsa and Gram Brown–Brenn histochemical stains were performed on selected pulmonary tissues to identify intralesional organisms.

### 2.2. Histopathology and Immunohistochemistry

All pulmonary tissues were initially screened to identify the typical histopathological patterns of interstitial pneumonia, bronchopneumonia, granulomatous pneumonia, and embolic pneumonia [25]. Thereafter, the selected pulmonary tissues were reviewed for predetermined histopathologic patterns of pulmonary disease or histological alterations and categorized as: 0, normal lung; 1, circulatory, reversible, and irreversible cellular alterations; 2, interstitial pneumonia; and 3, suppurative bronchopneumonia, as previously described [15]. Additionally, the histological elements identified in each pulmonary alteration were observed and tabulated.

Subsequently, pulmonary tissues were submitted to IHC assays designed to identify specific agents known to be associated with BRD: BVDV, BoAHV1, BRSV, BPIV-3, and *M. bovis* [4,10], as previously described [10]. These agents were selected due to the availability of monoclonal and/or polyclonal antibodies; a list of the antibodies used in this study with the respective dilutions and methods of antigen retrieval is provided (Table 1).

Positive controls consisted of pulmonary fragments known to contain antigens of BVDV, BoAHV1, BRSV, BPIV-3, and *M. bovis* [10]. Negative control was performed by replacing the primary antibody with diluent; positive and negative controls were included in each IHC assay. The data obtained were tabulated and analyzed.

### 2.3. Molecular Detection of Agents Associated with Reproductive Diseases in Cattle

The extracted nucleic acids from the lungs of fetuses number 9, 12, 17, 19, 23, 24, and 27–32 were used in molecular assays designed to identify a panel of infectious agents associated with reproductive disease in cattle, using PCR and/or RT-PCR assays as previously described [26] in a thermocycler (Proflex PCR System, Applied Biosystems; Marsiling Ind Estate Road 3, Singapore). These included PCR/RT-PCR assays to detect BoAHV1 [27], BVDV [28], *Listeria monocytogenes* [29], *Histophilus somni* [30], *Neospora caninum* [31], *Leptospira* spp. [32], and *Brucella abortus* [33]. Additionally, the extracted nucleic acids of these fetuses maintained at −80 °C (except numbers 9 and 12) were used in nested-PCR (nPCR) assays designed to amplify the bovine gammaherpesvirus 6 (BoGHV6) polymerase gene [34], since there is emerging evidence that BoGHV6 may be a potential pathogen of bovine fetuses [35]. Only fetuses submitted frozen and/or refrigerated were used for molecular detection; all other fetuses were submitted in formalin solution for histopathologic evaluation and, thus, were not used for molecular identification.

Positive controls consisted of nucleic acids extracted from Madin–Darby bovine kidney cell culture inoculated with BVDV (NADL strain) and BoAHV1 (Los Angeles strain) and field strains of previous cases of OvGHV2 [36], *L. monocytogenes* [37], *H. somni* [38], *N. caninum* [31], *Leptospira* spp., *B. abortus* [39], and BoGHV6 [35]. Nuclease-free water (Invitrogen Corp., Carlsbad, CA, USA) was used as negative control in all PCR and RT-PCR assays; positive and negative controls were included in all molecular assays. PCR/RT-PCR products were resolved by electrophoresis in 2% agarose gels, stained with ethidium bromide, and examined under ultraviolet light.

### 2.4. Sequencing and Phylogenetic Analysis of BoHV-6 Polymerase Gene

The nPCR products of BoGHV6 nPCR assays were purified using the PureLink^®^ Quick Gel Extraction and PCR Purification Combo Kit (Invitrogen^®^ Life Technologies, Carlsbad, CA, USA), quantified by using a Qubit^®^ Fluorometer (Invitrogen^®^ Life Technologies, Eugene, OR, USA), and submitted to sequencing in both directions with the forward and reverse primers used in the respective molecular assays in an ABI3500 Genetic Analyzer sequencer with the BigDye Terminator v3.1 Cycle Sequencing Kit (Applied Biosystems^®^, Foster City, CA, USA).

Sequence quality analyses and consensus sequences were obtained using PHRED and CAP3 software (http://asparagin.cenargen.embrapa.br/phph/, accessed on 21 April 2022), respectively. Similarity searches of the BoGHV6 polymerase gene were performed with nucleotide (nt) sequences deposited in GenBank using the Basic Local Alignment Search Tool software (https://blast.ncbi.nlm.nih.gov/Blast.cgi, accessed on 21 April 2022). Sequencing was done only for BoGHV6 since there is still controversy as to the role of this pathogen in infections [35].

### 2.5. Animal Ethics

This study followed the animal use rules of the National Council for Animal Control in Experiments and was approved by the Ethics Committee on Animal Usage, Universidade Estadual de Londrina (CEUA/UEL; protocol, 835.2019.45).

## 3. Results

### 3.1. Histopathological Findings and Pulmonary Patterns

During the period (2009–2019), 45 fetuses were submitted for routine post-mortem evaluations at UEL, southern Brazil, and three at UFMG, midwestern Brazil. However, only 34 fetuses from UEL, Paraná, and those from UFMG (n = 3), fulfilled the selection criteria and were included since they contained paraffin blocks, pulmonary tissues, and the biological data of the submitted animal.

**Figure 1:**
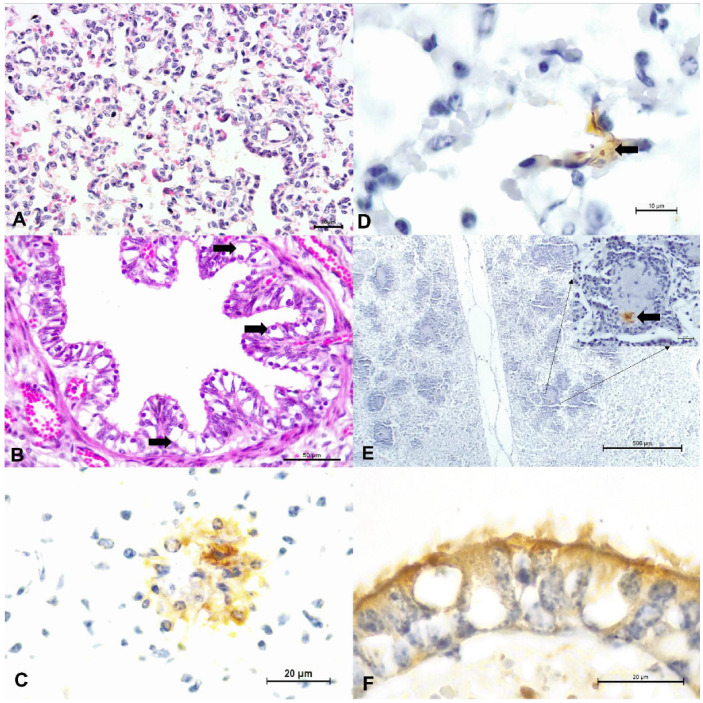
Principal histopathologic and immunohistochemical findings observed in fetal lungs of cattle. There is interstitial pneumonia (**A**) and degeneration (arrows) of bronchial epithelium (**B**). Observe positive intracytoplasmic immunoreactivity to antigens of BRSV (**C**), BVDV within alveolar epithelium (**D**), and within a region of suppurative bronchopneumonia (**E**); BVDV immunoreactivity is highlighted at the insert. There is positive intracytoplasmic immunoreactivity to BoAHV1 within degenerated bronchial epithelium (**F**). (**A**,**B**), Hematoxylin and eosin stain; (**C**–**F**), immunoperoxidase counterstained with Hematoxylin. Bars, (**A**,**C**,**F**), and insert, 20 µm; (**B**), 50 µm; (**D**), 10 um; (**E**), 500 µm.

Normal pulmonary tissue was observed in 48.8% (18/37) of the cases, circulatory and/or cellular alterations were diagnosed in 18.9% (7/37), 29.7% (11/37) had interstitial (Figure 1A), with one case (1/37; 2.7%) of fetal suppurative bronchopneumonia being identified (Table 2). Accordingly, pneumonia was identified in 35.1% (12/37) of the fetal tissues, while 64.9% (25/37) of the fetal lungs did not show histologic evidence of pneumonia.

The histological changes (n = 52) observed in the lungs evaluated with and without pneumonia are presented in Table 3; since some of these alterations occurred simultaneously in the same fetal lung. The most frequent histological change was pulmonary congestion (27%; 14/52), followed by ballooning degeneration (Figure 1B) of the bronchial epithelium (19.2%; 10/52) and bronchiolar (13.5%; 7/52). Additionally, the fetus with suppurative bronchopneumonia contained accumulations of an intralesional Gram-positive coccoid bacteria.

### 3.2. Relationship between Immunohistochemical Identification of Infectious Agents and Pulmonary Alterations

Table 4 demonstrates the relationship between pulmonary changes and IHC detection of intralesional agents. Positive immunoreactivity to antigens associated with BRD were observed in 29.7% (11/37) of the fetal lungs in the above-mentioned categories: 0, normal lung (n = 0); 1, pulmonary tissue with circulatory alterations (n = 5); 2, interstitial pneumonia (n = 6); and 3, suppurative bronchopneumonia (n = 1); 70.3% (26/37) of the fetal pulmonary fragments did not contain any of the analyzed agents. The most frequent intralesional agents identified was BRSV (16.2%; 6/37), followed by BVDV (13.5%; 5/37), BoAHV1 (8.1%; 3/37), *M. bovis* (5.4%; 2/37), and BPIV-3 (2.7%; 1/37).

**Figure 2:**
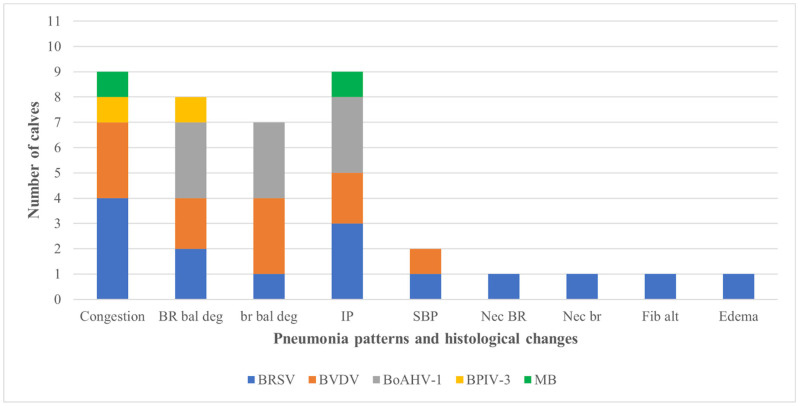
Relationship between pneumonic patterns and histological changes with the associated infected agent identified by immunohistochemistry in the lungs of aborted bovine fetuses. Footnote: BoAHV1: bovine alphaherpesvirus 1; BPIV-3: bovine parainfluenza virus 3; BRSV: bovine respiratory syncytial virus; BVDV: bovine viral diarrhea virus; *M. bovis: Mycoplasma bovis.* IP, interstitial pneumonia; BR bal deg, bronchial epithelial ballooning degeneration; SBP, suppurative bronchopneumonia; br bal deg, bronchiolar epithelial ballooning degeneration; BR nec, bronchial epithelial necrosis; br nec, bronchiolar epithelial necrosis; Fib Alt, fibrinoid alteration.

The association between intralesional accumulation of tissue antigens and the categories of pulmonary alterations identified in the fetal lungs by IHC is graphically presented (Figure 2). All antibodies used showed patchy, intracytoplasmic immunoreactivity within several histologic pulmonary elements. Interstitial pneumonia was associated with intralesional antigens of BRSV (n = 3), BoAHV1 (n = 3), BVDV (n = 2), and *M. bovis* (n = 1). However, in 46.2% (6/13) of lungs with interstitial pneumonia, none of the agents investigated was identified. The suppurative bronchopneumonia observed in fetus #37 contained intralesional antigens of BVDV and BRSV, with accumulations of Gram-positive bacteria. Circulatory (category 1) alterations were associated with the intralesional accumulations of antigens of BRSV (n = 3), BVDV (n = 2), BPIV-3, and *M. bovis* (n = 1).

Positive immunoreactivity to BRSV antigens was observed within degenerated and normal bronchial and bronchiolar epithelial cells, alveolar epithelium, and epithelial cells of the mixed bronchial gland (Figure 1C). Positive immunoreactivity to BVDV was observed within normal and degenerated bronchial epithelial cells and also within the suppurative exudate of a fetus with bronchopneumonia (Figure 1D,E). Intralesional BoAHV1 antigens were detected within normal bronchial and bronchiolar epithelial cells, chondrocytes of hyaline cartilage, and capillary endothelium (Figure 1F). Infections due to *M. bovis* resulted in positive immunolabelling within normal, degenerated, and necrotic bronchial and bronchiolar epithelial cells, necrotic epithelial cells of the mixed bronchial glands, endothelial cells, and alveolar macrophages. BPIV-3 antigens revealed positive intracytoplasmic immunoreactivity within normal bronchiolar epithelial cells and alveolar macrophages.

### 3.3. Molecular Identification of Pathogens and Relationship with Histologic Pulmonary Alterations

The results of the molecular detection of a panel of reproductive disease agents and BoGHV6 from the lungs of the fetuses are presented in Table 3, with at least one infectious agent being amplified from most (75%; 9/12) of the fetuses evaluated. The agents detected were *Leptospira* spp., (n = 3), BVDV (n = 2), *N. caninum* (n = 2*)*, and *B. abortus* (n = 2), while BoGHV6 was identified in the lungs of 4 fetuses. Sequence analysis of the amplicons confirmed the identity of the products obtained from the BoGHV6 nPCR assay.

Furthermore, *Leptospira* spp. was the only agent identified in a lung (#9) without pneumonia and was also observed in a fetal lung with interstitial pneumonia that was concomitantly infected by *B. abortus*. Additionally, BVDV, *N. caninum*, and *B. abortus* were associated with pulmonary congestion (Table 3). Additionally, 2 (#24 and 32) of the 4 fetal lungs infected by BoGHV6 had a histologic diagnosis of interstitial pneumonia, with 1 lung (#32) being simultaneously infected by BRSV and BoAHV1 as demonstrated by IHC, while the other fetal lung (#24) was only infected by BoGHV6, suggesting a possible association between interstitial fetal pneumonia and BoGHV6.

Moreover, when all IHC and molecular analyses were considered, single (n = 7), dual (n = 3), triple (n = 4), and quadruple (n = 1) infections were identified. Alternatively, tissue antigens and/or nucleic acids were not identified in 59.5% (22/37) of the fetal lungs. Single fetal infections were associated with BRSV, BVDV (n = 2), *Leptospira* spp., BoAHV1, and BoGHV6 (n = 1). The only quadruple infection identified was associated with the simultaneous identification of antigens of BRSV and *M. bovis* by IHC and the nucleic acids of *N. caninum* and BoGHV6 via PCR in a congested fetal lung (#27).

## 4. Discussion

This study demonstrates the presence of pathogens known to produce respiratory diseases in cattle within the lungs of aborted bovine fetuses, amplified BoGHV6 DNA from some of these, and associates these pathogens with or without pulmonary alterations in bovine fetuses, demonstrating clear evidence that these fetuses were infected [24]. Furthermore, some well-known pathogens of reproductive disease in cattle, such as BVDV, BoAHV1, *Leptospira* spp., *B. abortus*, and *M. bovis,* were also identified and associated with pulmonary alteration(s), collaborating with the results of previous studies that have demonstrated *B. abortus* [19,20], *M. bovis* [17], BoAHV1 [16,21], BVDV [10] in the fetal lungs of cattle. Collectively, fetal infection by these pathogens was probably due to intrauterine infection resulting from transplacental transmission [40,41]. Consequently, most of the disease agents herein identified may be classified either as primary or secondary fetopathic agents of cattle [24,40] and could have produced direct or indirect effects on fetal survival [41]. The primary fetopathy agents herein identified (BVDV, BoAHV1, *N. caninum*, *B. abortus*, and *Leptospira* spp.) can cross the placental barrier, producing fetal disease in healthy cows, whereas the secondary or opportunistic agent (*H. somni*) crosses the placental barrier when there is placental damage, alteration to the microflora of the reproductive tract, or in immunocompromised cows [40].

The novelty of this study is the identification of antigens of two classical respiratory pathogens (BRSV and BPIV-3) within fetal lungs, the association of BRSV with interstitial pneumonia in two fetuses, while there was double infection by these viruses in one congested fetal lung. As far as the authors are aware, this is a novel description of BRSV-associated lesions in bovine fetuses, while there is a previous report of the participation of BPIV-3 in the development of fetal interstitial pneumonia in cattle [18].

Furthermore, the amplification of BoGHV6 DNA from multiple fetuses with interstitial pneumonia and other pulmonary alterations supports the theory that this pathogen should be considered an agent of fetal disease in cattle [35]. This is supported by previous studies which have identified this virus in an aborted fetus from Canada [42], within uterine secretions of a cow from Belgium [43], and in cows with endometritis from the UK [44]. Recently, we have identified BoGHV6 in several bovine fetuses that were simultaneously infected with *H. somni* and other well-known pathogenic abortive agents of cattle, while one fetus with myocarditis contained only BoGHV6 DNA [36]. Collectively, these are adequate emerging evidence to suggest a possible association of BoGHV6 with fetal disease and possible fetal death in cattle [35]. Furthermore, BoGHV6 and BRSV should be added to the list of secondary fetopathic agents of cattle [40] until the direct and/or indirect effects of these infections on fetal survival [41] and the associated pathogenesis with possible disease manifestations are fully investigated. Nevertheless, the IHC detection of tissue antigens of BoGHV6, using monoclonal or polyclonal antibodies, would definitely determine the participation of this pathogen in the development of fetal pneumonia. Consequently, it would be interesting to determine whether BoGHV6 is just an innocent bystander or an inductor of fetal disease in cattle [35] since this pathogen was frequently identified in mixed infections during this investigation. However, the current role of BoGHV6 in the development of disease is unknown, so caution must be used in the interpretation of these results until experimental studies have demonstrated the participation of this pathogen in the development of disease processes.

This study used two methods to associate the pathogens with infection: molecular and IHC detection/identification. Although molecular detection by PCR/RT-PCR is more sensitive than IHC, intralesional detection of these pathogens by IHC is strong evidence of their association with the disease process [23]. Molecular detection of an infectious agent should not be definitively interpreted as the cause of a specific disease process but is fundamental to differentiate between vaccine and field strain of disease agents [22]. Additionally, the IHC identification and molecular detection of tissue antigens and nucleic acids, respectively, with related histologic evidence of disease in fetal tissues, are suggestive of causal association [40]. Diagnostic IHC was used in previous studies to effectively associate intralesional organisms within fetal lungs of cattle [10,16,17], and as indicated previously, would be needed to definitely associate BoGHV6 with fetal pathology.

Most of the immunohistochemical findings associated with BRD pathogens herein identified in the fetal lungs were previously observed in the lungs of feedlot and dairy cattle with histological evidence of several patterns of pulmonary disease [10,15]. Intralesional immunoreactivity for *M. bovis* was observed within several epithelial cells of the lung; a previous investigation using IHC demonstrated positive immunoreactivity with the epithelial cells of the alveolar wall but with multifocal identification of *M. bovis* proteins by ISH [17]. Collectively, these results suggest that the distribution of *M. bovis* antigens and/or proteins within the lungs of bovine fetuses seems to be multifocal and not restricted to a specific histologic element of the lung. However, the classical pulmonary pattern of necrosuppurative or suppurative bronchopneumonia associated with pulmonary infections due to *M. bovis* [10] was not observed during this study when compared to a previous report of *M. bovis*-associated pulmonary diseases in a fetus [17]. The fetuses herein infected by *M. bovis* had histologic evidence of interstitial pneumonia and pulmonary congestion and contained other disease pathogens, including *N. caninum*, BVDV, and BoAHV1, suggesting that *M. bovis* may not have been directly related to the development of the principal pattern of pulmonary disease identified in these fetuses.

During this study, single, double, triple, and quadruple infections were observed by the identification of intralesional antigens and/or nucleic acids of primary and secondary agents. Single infections were predominant, as frequently described in fetal deaths related to infectious diseases [40], and also occurred in other studies investigating fetal pulmonary diseases of cattle [17,18,19]. Alternatively, the multiple concomitant infections identified in this study are not frequently identified in fetal pathology [40], with only one previous description of spontaneous dual infections in bovine fetuses [16]. Although the reason for the identification of mixed infections in bovine fetuses is unclear, high environmental infectious challenges, such as the endemicity of a disease pathogen, were proposed [40]. Pathogen endemicity seems plausible to the dynamics of reproductive disease pathogens in Brazil, considering that BVDV, BoAHV1, *Leptospira* spp., *B. abortus* [26,45,46]*,* and to some extent *H. somni* [26,47], are frequently associated with fetal pathology and/or abortions in cattle from this country. Additionally, the frequency of concomitant fetal pulmonary infections herein identified may suggest that simultaneous infections in bovine fetuses may be more common than previously described [40].

## 5. Conclusions

In conclusion, molecular and IHC detection confirmed the presence of several agents associated with pulmonary and reproductive diseases of cattle within fetal lungs that had histologic evidence of interstitial pneumonia and/or pulmonary alterations. Collectively, these assays have demonstrated the occurrence of primary and secondary fetopathy agents in these fetuses and indicate intrauterine/transplacental infection. The amplification of BoGHV6, BRSV, and BPIV-3 from the lungs of several fetuses with histologic evidence of pulmonary alteration, particularly with interstitial pneumonia, suggests that these pathogens should be considered as putative fetopathy agents of cattle.

Nevertheless, the role of BoGHV6 in the development of disease processes is not fully known and must be confirmed by experimental and in situ studies. Finally, the relative frequency of simultaneous infections herein identified may indicate that concomitant fetal infectious may be more frequent than previously diagnosed.

## Figures and Tables

**Table 1:** Antibodies, dilutions, and antigen retrieval methods used in immunohistochemical assays.

Antibody (Clone)	Antigen Retrieval	Dilution	Distilled Water (mL)	Hydrogen Peroxide (6%; mL)	Source
BoAHV1 (MAb 9E7)	Citrate buffer (pH 6)	1:700	50	100	VMRD, Pullman, WA, USA
BPIV-3	TRIS+EDTA buffer (pH 9)	1:30	110	40	Dr. Eduardo F. Flores
BRSV (15c7)	Citrate buffer (pH 6)	1:300	50	100	Dr. Eduardo F. Flores
BVDV (15c5)	Citrate buffer (pH 6)	1:1500	50	100	Dr. Eduardo F. Flores
*Mycoplasma bovis*	Citrate buffer (pH 6)	1:10	110	40	Dr. Lucienne G. Pretto-Giordano

BoAHV1: bovine alphaherpesvirus 1; BPIV-3: bovine parainfluenza virus 3; BRSV: bovine respiratory syncytial virus; BVDV: bovine viral diarrhea virus; MAb: monoclonal antibody; TRIS: tris (hydroxymethyl) aminomethane; EDTA: ethylenediaminetetraacetic acid.

**Table 2:** Patterns of pulmonary disease observed in fetuses from southern and midwestern Brazil.

Histological Pattern of Pneumonia	Number of Fetuses	Frequency (%)
Interstitial pneumonia	12	32.4
Suppurative bronchopneumonia	1	2.7
Without pneumonia	24	64.9
Total	37	100

**Table 3:** Histological findings observed within the patterns of pulmonary lesions observed in 37 fetuses.

Histological Findings	Number of Fetuses	Frequency (%)
Pulmonary congestion	14	27
Normal lung	14	27
Ballooning degeneration of the bronchial epithelium	10	19.2
Ballooning degeneration of the bronchiolar epithelium	7	13.5
Alveolar edema	3	5.8
Bronchial epithelial necrosis	2	3.8
Bronchiolar epithelial necrosis	1	1.9
Fibrinoid alteration	1	1.9
Total	52	100

**Table 4:** Principal histopathological findings, patterns of pneumonia, and infectious agents observed in the lungs of bovine fetuses by immunohistochemistry and molecular detection.

Fetuses	Principal Histopathologic Alteration /Pattern of Pulmonary Disease	Immunohistochemistry	Molecular Detection		**Type of Infection**
			Reproductive Panel	BoGHV6	
Lungs without any infectious disease agent by IHC					
#1	Normal	−ve	ND	ND	None
#2	Normal	−ve	ND	ND	None
#3	Normal	−ve	ND	ND	None
#4	Normal	−ve	ND	ND	None
#5	Normal	−ve	ND	ND	None
#6	Normal	−ve	ND	ND	None
#7	Normal	−ve	ND	ND	None
#8	Normal	−ve	ND	ND	None
#9 *	Normal	−ve	*Leptospira* spp.	−ve	Single
#10	Normal	−ve	ND	ND	None
#11	Normal	−ve	ND	ND	None
#12 *	Normal	−ve	−ve	−ve	None
#13	Normal	−ve	ND	ND	None
#14	Normal	−ve	ND	ND	None
#15	Congestion	−ve	ND	ND	None
#16	Congestion	−ve	ND	ND	None
#17 *	Congestion	−ve	BVDV	−ve	Single
#18	Congestion	−ve	ND	ND	None
#19 *	Congestion Ballooning degeneration (bronchial epithelium)	−ve	−ve	−ve	None
#20	Congestion Edema Ballooning degeneration (bronchial and bronchiolar epithelium)	−ve	ND	ND	None
#21	Congestion Interstitial pneumonia	−ve	ND	ND	None
#22	Congestion Edema Interstitial pneumonia	−ve	ND	ND	None
#23 *	Interstitial pneumonia	−ve	*Leptospira* spp. *B. abortus*	−ve	Dual
#24 *	Interstitial pneumonia	−ve	−ve	+ve	Single
#25	Interstitial pneumonia	−ve	ND	ND	None
#26	Interstitial pneumonia Ballooning degeneration (bronchial epithelium) Necrosis of bronchial epithelium	−ve	ND	ND	None
Lungs with infectious agent(s) identified by IHC					
#27 *	Congestion	BRSV, *M. bovis*	*N. caninum*	+ve	Quadruple
#28 *	Congestion Ballooning degeneration (bronchial epithelium)	BRSV, BPIV-3	*N. caninum*	−ve	Triple
#29 *	Congestion Ballooning degeneration (bronchial and bronchiolar epithelium)	BVDV	*B. abortus*, BVDV	+ve	Triple
#30 *	Congestion Ballooning degeneration (bronchial and bronchiolar epithelium)	BVDV	−ve	−ve	Single
#31 *	Interstitial pneumonia Ballooning degeneration (bronchial and bronchiolar epithelium)	BVDV	*Leptospira* spp.	−ve	Dual
#32 *	Interstitial pneumonia Ballooning degeneration (bronchial and bronchiolar epithelium)	BRSV, BoAHV1	−ve	+ve	Triple
#33	Interstitial pneumonia Ballooning degeneration (bronchial and bronchiolar epithelium)	BoAHV1	ND	ND	Single
#34	Interstitial pneumonia Ballooning degeneration (bronchial and bronchiolar epithelium)	BVDV, BoAHV1, *M. bovis*	ND	ND	Triple
#35	Interstitial pneumonia Fibrinoid alteration Necrosis of bronchial and bronchiolar epithelium	BRSV	ND	ND	Single
#36	Interstitial pneumonia Edema Congestion	BRSV	ND	ND	Single
#37	Congestion Suppurative bronchopneumonia Accumulation of bacteria	BVDV, BRSV	ND	ND	Dual

−ve, negative; +ve, positive; ND, not done. *, fetuses evaluated for the molecular identification of a panel of reproductive disease agents (BoAHV1, BVDV, *Leptospira* spp., *Histophilus somni*, *Brucella abortus*, *Neospora caninum*, and *M. bovis*). BoAHV1: bovine alphaherpesvirus 1; BPIV-3: bovine parainfluenza virus 3; BRSV: bovine respiratory syncytial virus; BVDV: bovine viral diarrhea virus; *M. bovis*: *Mycoplasma bovis*.

## Data Availability

Details of all molecular sequences identified during this study are deposited in GenBank.
